# Anti-inflammatory effects of isoketocharbroic acid from brown alga, Sargassum micracanthum

**DOI:** 10.17179/excli2015-555

**Published:** 2015-10-15

**Authors:** Young Min Ham, Weon-Jong Yoon, Wook Jae Lee, Sang-Cheol Kim, Jong Seok Baik, Jin Hwa Kim, Geun Soo Lee, Nam Ho Lee, Chang-Gu Hyun

**Affiliations:** 1 Jeju Biodiversity Research Institute (JBRI), Jeju Technopark, Jeju 699-943, Korea; 2 Nakdonggang National Institute of Biological Resources, Chungbuk 742-350, Korea; 3 Cosmetic Science Center, Department of Chemistry and Cosmetics, Jeju National University, Jeju 690-756, Korea; 4 R & D Center, Hanbul Cosmetics Co., Chungbuk 369-830, Korea

**Keywords:** brown alga, inflammation, isoketochabrolic acid, Sargassum micracanthum

## Abstract

During our on-going screening program designed to isolate natural compounds from marine environments, we isolated isoketochabrolic acid (IKCA) from *Sargassum micracanthum*, an important brown algae distributed in Jeju Island, Korea. Furthermore, we evaluated the inhibitory effects of IKCA on nitric oxide (NO) production in lipopolysaccharide (LPS)-triggered macrophages. IKCA strongly inhibited NO production, with an IC_50_ value of 58.31 μM. Subsequent studies demonstrated that IKCA potently and concentration-dependently reduced prostaglandin E_2_ (PGE_2_), tumor necrosis factor-alpha (TNF-α), interleukin (IL)-1β, and IL-6 cytokine production. In conclusion, to the best of our knowledge, this is the first study to show that IKCA isolated from *S. micracanthum* has a potent anti-inflammatory activity. Therefore, IKCA might be useful as an anti-inflammatory health supplement or functional cosmetics.

## Introduction

The inflammatory response is a highly regulated defense process that is activated in response to a wide variety of exogenous and endogenous stimuli, such as pathogens, damaged cells, or irritants. Normally, this process attempts to remove the injurious stimuli and promotes wound healing (Choi et al., 2014[4]; Guo et al., 2015[6]). However, an excessive inflammatory response has been recognized as one of the principal causes of chronic inflammation, such as allergy, asthma, rheumatoid arthritis, Alzheimer's disease (AD), cardiovascular disease, and even cancer (Kim et al., 2014[11]; Dutta and Das, 2016[5]; Schuliga, 2015[17]). Chronic inflammation is caused by pro-inflammatory mediators, which include both pro-inflammatory cytokines, such as tumor necrosis factor-α (TNF-α), interleukin-1β (IL-1β), and interleukin-6 (IL-6); and pro-inflammatory factors like nitric oxide (NO) and prostaglandin E_2_ (PGE_2_). These pro-inflammatory mediators are key regulators of immune responses and are therefore potential targets for the development of new therapeutic strategies (Moon et al., 2011[15]; Kim et al., 2013[12]; Choi et al., 2014[4], 2015[3]). A variety of inflammatory diseases are immedicable, but steroidal or non-steroidal anti-inflammatory drugs (NSAIDs) have been developed and are widely used for relieving inflammatory symptoms. However, long-term treatment of these drugs produces serious side effects, including cardiovascular complications, immunodeficiency diseases, and gastrointestinal disorders (Cai et al., 2014[1]; Choi et al., 2015[3]; Hyun et al., 2015[8]). For the reasons above, research efforts have focused on the identification of new anti-inflammatory agents with few side effects from natural products, including phytochemicals and algae-derived products (Kim et al., 2013[13], 2014[10]; Yang et al., 2013[20][21], 2014[22]).

The marine ecosystem is a rich resource with the potential to produce a wide variety of bioactive and/or secondary metabolites, such as bacterial polyketides, phlorotannins, plastoquinones, sterols, and chromenes, with a particular significance for multiple nutraceutical, cosmeceutical, and pharmaceutical applications. Among marine organisms, algae are a relatively unexplored source of protein, iodine, vitamins, minerals, and secondary metabolites that might be promising anti-inflammatory agents. Indeed, a recent functional cosmetic ingredient research showed that marine algae are a rich source of cosmeceuticals with a wide range of biological activities (Chinnababu et al., 2015[2]; Nitschke and Stengel, 2015[16]).

*Sargassum micracanthum *(Kützing) Endlicher, a brown alga, is distributed worldwide, ranging from temperate to subtropical regions. It is a representative seaweed that can easily be collected at Jeju Island, Korea. Studies have been conducted on anti-bacterial, anti-obesity, anti-inflammatory, and anti-oxidation activities of *S. micracanthum* (Jeong et al., 2013[9]; Lee, 2014[14]). In addition, we reported that fucosterol, sargaquinoic acid, and sargachromenol, isolates from *S. micracanthum*, showed anti-inflammatory and/or anti-oxidant activities *in vitro* (Ham et al., 2010[7]; Yang et al., 2013[20]). As part of our continuing efforts directed toward the discovery of structurally interesting and biologically active anti-inflammatory agents from micro algae, we isolated isoketochabrolic acid (IKCA) from *S. micracanthum*, using activity-directed fractionation. In this study, we characterized the structural identity of IKCA using spectroscopy (1H proton-nuclear magnetic resonance (1H NMR) and 13C NMR). In addition, as an initial screen to determine if IKCA exhibits any anti-inflammatory activity, we evaluated whether IKCA inhibited the production of the pro-inflammatory mediators NO and PGE_2_ and the cytokines TNF-α, IL-1β, and IL-6 in lipopolysaccharide (LPS)-stimulated macrophages.

## Material and Methods

### Extraction and isolation of isoketochabrolic acid

**Figure 1:**
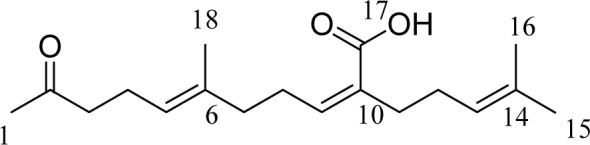
The structure of isoketochabrolic acid (IKCA)

*S. micracanthum *was collected from the coasts of Chuja Island (dependent islets of Jeju Island) in May 2009 and verified by Dr. Wook Jae Lee at Jeju Biodiversity Research Institute (JBRI). *S. micracanthum *were washed three times with tap water to remove any salt, sand, and epiphytes attached to their surface. They were dried at 60 °C for 24 h in an oven and pulverized in a grinder prior to extraction. The dried powder (370 g) was extracted with 70 % aqueous ethanol with stirring for 2 days at room temperature. The filtrate was concentrated under reduced pressure. The extract (36 g) was suspended in water (4.0 L) and successively partitioned into *n*-hexane, methylene chloride, ethyl acetate, and *n*-butanol fractions. The methylene chloride fraction (6.5 g) was dissolved in solvent, mixed with celite (60 g), and evaporated using a rotary vacuum evaporator. After concentration, it was chromatographed and eluted using the solvents (500 ml) *n*-hexane (2L), *n*-hexane/methylene chloride (10:1, 5:1, 2:1, 2L respectively), methylene chloride (2L), methylene chloride/ethyl acetate (10:1, 5:1, 2:1, 2L respectively), ethyl acetate (2L), and methanol (2L) in order. The fifth fraction (700 mg) from the celite column was subjected over a silica gel column using n-hexane:EtOAc (3:1) in order to obtain 12 sub-fractions (F-1 to F-12). All fractions containing the same constituents identified on the thin layer chromatography (TLC) plates were combined, and the solvents were evaporated using a rotary vacuum evaporator. Structures of fraction 9 (F9, 3.5 mg) were determined using 1H NMR and 13C NMR. The compound's structural identity was determined by one- and two-dimensional NMR spectroscopic analysis and comparison to published values. Structures of these compounds are shown in Figure 1(Fig. 1). 

### Chemicals and reagents

Dulbecco's modified Eagle's medium (DMEM), foetal bovine serum (FBS), penicillin, and streptomycin for cell culture were obtained from Invitrogen-Gibco (Carlsbad, CA, USA). Mouse TNF-α, IL-1β, IL-6, and PGE_2_ enzyme-linked immunosorbent assay (ELISA) kits were purchased from R&D Systems (Minneapolis, MN, USA) and Cayman Chemicals (Ann Arbor, MI, USA). All other reagents were purchased from Sigma-Aldrich Chemical Co. (St. Louis, MO, USA).

### RAW 264.7 cell culture

Macrophage cells were cultured as described previously with slight modification (Kim et al., 2013[12][13]). The RAW 264.7 macrophage cell line, obtained from the Korean Cell Line Bank (KCLB; Seoul, Korea) was maintained in DMEM supplemented with 10 % heat-inactivated FBS, 3 mM glutamine, antibiotics (100 U/ml penicillin and 100 U/ ml streptomycin) in a 95 % air, 5 % CO_2_ humidified atmosphere at 37 °C. Cells subjected to no more than 20 passages were used for the experiments and subcultured every 2-3 days. In all experiments, the RAW 264.7 cells were incubated in the presence or absence of various concentrations of IKCA, which was always added 1 h prior to LPS (1 μg/ml) treatment.

### Lactic acid dehydrogenase (LDH) cytotoxicity assay

Cell viability was measured as described previously with slight modification using the LDH cytotoxicity detection kit (Promega, Madison, WI), which quantifies the release of LDH from cells into the culture medium (Yoon et al., 2010[23]). Macrophage cells (1.5 × 10^5^ cells/mL) were seeded in 96 well plates, pre-incubated for 18 h, and then treated with LPS (1 μg/mL) plus IKCA (12.5 to 100 μM) at 37 °C for 24 h. Supernatants from the cultures were collected and used in the LDH assay, as instructed by the manufacturer. The optical density of the solution at a wavelength of 490 nm was measured using a microplate reader (Power Wave, Bio-Tek Inc., Winooski, VT). Percent cytotoxicity was determined relative to the control group. All experiments were performed in triplicate.

### NO determination

Nitrate concentration was measured as described previously with slight modification using the Griess reagent (Kim et al., 2013[12][13]). RAW 264.7 cells were seeded at 1.5×10^5^ cells/well in 24-well plates at 37 °C in a humidified atmosphere containing 5 % CO_2_ overnight. After 18 h, the cells were treated with various concentrations (12.5, 25, 50, and 100 μM) of IKCA for 1 h and then LPS (1 μg/mL) was added for 24 h. NO levels were determined by measuring nitrite levels using the supernatant (100 μL) mixed with the same volume of Griess reagent (1 % sulphanilamide and 0.1 % N-[1-naphthyl]-ethylenediamine dihydrochloride in 5 % phosphoric acid %) for 10 min, and absorbance was then measured at 540 nm using a microplate reader. Fresh culture media were used as blanks in all experiments. The nitrite concentration in samples was read off a standard curve of sodium nitrite.

### Inflammatory cytokine assay

ELISA was used to determine the inhibitory effects of different concentrations (25, 50, and 100 μM) of IKCA on the production of PGE_2_, TNF-α, IL-1β, and IL-6 in LPS-treated RAW 264.7 cells. RAW 264.7 cells were stimulated for 24 h, and the supernatant was harvested and assayed according to the manufacturer's instructions for the relevant ELISA kit. Standards were prepared from recombinant mouse cytokines separately (R&D). Results from three independent experiments were used for statistical analysis.

### Statistical analysis

All data are expressed as means ± standard deviation (S.D.). Significant differences among the groups were determined using the unpaired Student's *t*-test. A value of *P<0.05 was considered to be statistically significant.

## Results and Discussion

Previously, we isolated sargachromenol, sargaquinoic acid and fucosterol from *S. micracanthum*, a brown algae, and identified their appreciable anti-oxidant effect (Ham et al., 2010[7]). One of them, sargachromenol, was also found to inhibit LPS-induced production of inflammatory mediators in RAW 264.7 cells (Yang et al., 2013[20]). In the present study, we isolated another active substance, IKCA, from *S. micracanthum* and examined its possible anti-inflammatory activity. The ethanol extract of *S. micracanthum* was suspended in sterile purified water and extracted successively with *n*-hexane, MeCl, EtOAc, and BuOH. The MeCl fraction was subjected repeatedly to column chromatography over celite and silica gel in various solvent systems to yield the active ingredient. It was identified as IKCA (Figure 1(Fig. 1)) by comparison of physical and spectroscopic data with published values (Su et al., 2007[18]). The 1H NMR and 13C NMR was as follows: 1H NMR (500 mHz, CDCl3) δH (ppm): 5.95 (1H, t, J=7.5Hz, C-9), 5.07 (2H, m, C-5, C-13), 2.56 (2H, t, J=7.5 Hz, C-8), 2.44 (2H, t, J=7.5 Hz, C-3), 2.24 (4H, m, C-4, C-11), 2.13 (3H, s, C-1), 2.10 (2H, t, J=7.5 Hz, C-7), 2.03 (2H, m, C-12), 1.66 (3H, s, C-15), 1.63 (3H, s, C-18), 1.62 (3H, s, C-16); 13C-NMR (125 MHz, CDCl3) δc (ppm): 209.1 (C-2), 172.1 (C-17), 145.3 (C-9), 135.8 (C-6), 132.5 (C-14), 130.8 (C-10), 123.6 (C-13), 123.5 (C-5), 43.9 (C-3), 39.2 (C-7), 34.8 (C-77), 30.1 (C-1), 28.3 (C-8), 28.1 (C-12), 25.9 (C-15), 22.6 (C-4), 17.9 (C-16), 16.1 (C-18).

**Figure 2:**
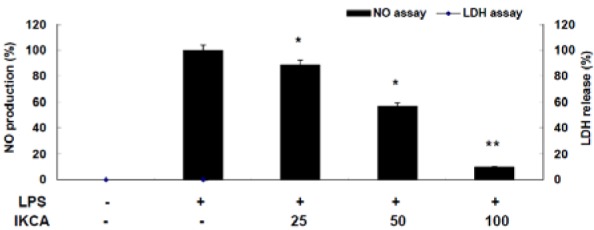
Effect of IKCA on nitric oxide production in lipopolysaccharide (LPS)-stimulated RAW264.7 cells. The cells (1.5 × 10^5^ cells/ml) were stimulated with 1 µg/mL of LPS only or with LPS plus various concentrations (25, 50, 100 µM) of IKCA for 24 h. Nitric oxide (NO) production was determined by the Griess reagent method. Cytotoxicity was determined using the lactic acid dehydrogenase (LDH) method. The data represent the mean ± S.D of triplicate experiments.*P<0.05, **P<0.01

Since LPS-stimulated macrophages produce a variety of pro-inflammatory mediators, such as NO, TNF-α, and IL-6, which are key regulators of immune responses, these mediators, therefore, may be an essential target for the development of anti-inflammatory materials (Waltz etal., 2015[19]) To demonstrate the anti-inflammatory activity of IKCA, we first assessed its ability to inhibit LPS-induced NO production in LPS-stimulated macrophage RAW 264.7 cells. RAW 264.7 cells were treated with various concentrations (25, 50, and 100 μM) of IKCA and cell viability was measured by LDH assay. As shown in Figure 2(Fig. 2), NO production increased >12 fold in LPS-activated macrophages relative to untreated macrophages. On the other hand, IKCA reduced LPS-induced NO production in a concentration-dependent manner (IC_50_ = 58.31 μM).

**Figure 3:**
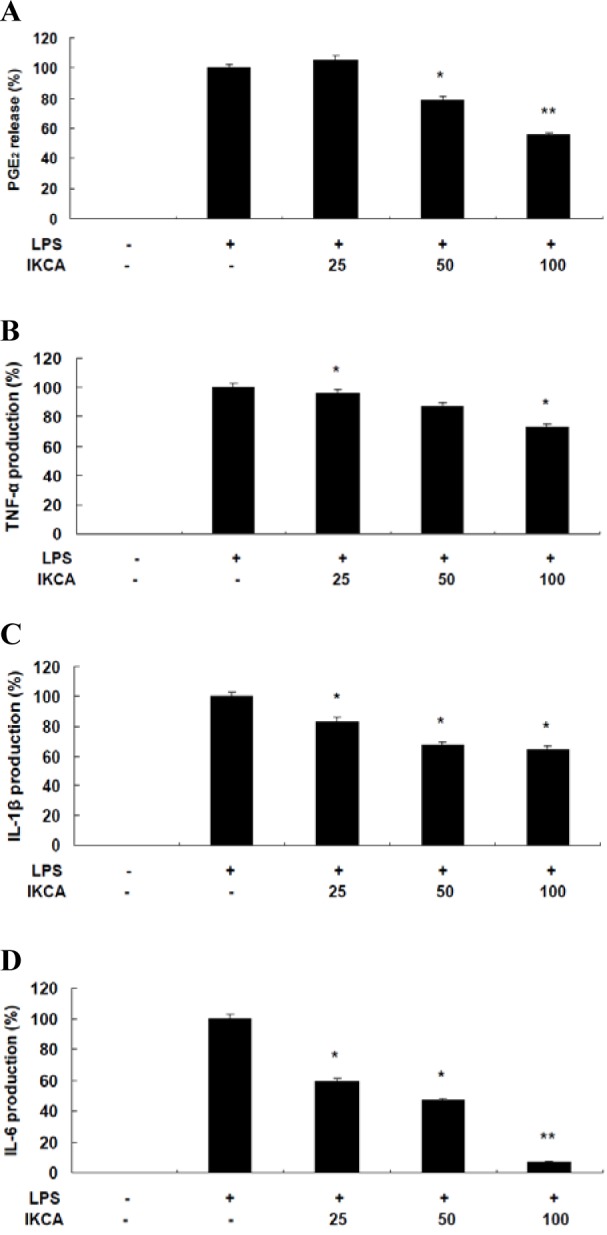
A,B: Effect of IKCA on PGE2 (A), TNF-‭‭⁯α (B) production in LPS-stimulated RAW 264.7 cells. C, D: Effect of IKCA on IL-1ß (C), and IL-6 (D) production in LPS-stimulated RAW 264.7 cells. The cells (1.5 × 10^5^ cells/ml) were stimulated with 1 µg mL^-1^ of LPS only or with LPS plus various concentrations (25, 50, 100 µM) of IKCA for 24 h. After 24 h, levels of PGE2, TNF-‭‭⁯α, IL-6, and IL-1ß in the culture supernatants were measured by an enzyme-linked immunosorbent assay (ELISA) kit. The data represent the mean ± SEM of triplicate experiments.*P<0.05, **P<0.01

No concentration (up to 50 μM) of IKCA displayed significant cytotoxicity, indicating that the anti-inflammatory effects of IKCA were not attributable to cytotoxicity. To further elucidate the anti-inflammatory mechanisms of IKCA, we measured the levels of PGE_2_, IL-6, IL-1β, and TNF-α in culture supernatants using ELISAs. Treatment of RAW 264.7 cells with LPS alone resulted in significant increase in cytokine production compared to the drug groups (Figure 3(Fig. 3)). However, the levels of NO, PGE_2_, IL-6, IL-1β, and TNF-α in the supernatants of LPS stimulated cells pretreated with 25, 50, and 100 μM IKCA were reduced significantly compared to the LPS group in a dose-dependent manner (* P < 0.05, ** P < 0.01) (Figure 3(Fig. 3)). In conclusion, the present study is, to the best of our knowledge, the first to isolate IKCA from *S. micracanthum* and to elucidate its anti-inflammatory properties, which was mediated through the suppression of NO, PGE_2_, IL-6, IL-1β, and TNF-α production in LPS-stimulated RAW 264.7 cells. These findings indicated that IKCA might be a promising agent for the clinical prevention and treatment of inflammation-associated human health in the future.

## Acknowledgements

This research was supported by the Industrial Development Program for Economic Cooperation Region (R0002894), which is managed by the Ministry of Trade, Industry & Energy, Korea. We are grateful to Jeju Technopark for providing research facilities for this study.
